# Community as a superpower: Why refugee youth need inclusion, education, and mentorship

**DOI:** 10.1371/journal.pmen.0000338

**Published:** 2025-06-18

**Authors:** Mohammad Yasir Essar, Gabriel E. Fabreau

**Affiliations:** Refugee Health YYC, O’Brien Institute for Public Health, Cumming School of Medicine, University of Calgary, Calgary, Alberta, Canada; PLOS: Public Library of Science, UNITED KINGDOM OF GREAT BRITAIN AND NORTHERN IRELAND

“*Refugees are mothers, fathers, sisters, brothers, and children, with the same hopes and ambitions as us—except that a twist of fate has bound their lives to a global refugee crisis on an unprecedented scale.*” — Khaled Hosseini

Today, over 122.6 million people have been forcibly displaced due to conflicts and disasters, with young people being disproportionately affected [1]. Crises in Gaza, Afghanistan, Ukraine, Syria, Sudan, and Myanmar exacerbate geopolitical tensions, economic instability, and climate-related disasters. Forcibly displaced youth (ages 15–24) face disrupted education, limited employment, and uncertain futures, significantly compromising not only their physical health, but also their mental health and emotional wellbeing [2].

One of us (YE), from Afghanistan, experienced these challenges firsthand, fleeing Kabul in August 2021, days before the Taliban’s takeover. In Tajikistan, he faced numerous hardships common to refugees displaced by war and conflict, including loss of community support, stigma, racism, discrimination from local authorities, and language barriers. These experiences erode refugee youths’ sense of identity, belonging and safety—factors known to critically impact mental health [3]. In Yasir’s case, language barriers hindered access to essential services, healthcare, education, and employment opportunities, while the constant fear of deportation back to Afghanistan intensified psychological distress. As highlighted by World Health Organization (WHO), these intersecting adversities place refugees at heightened risk of anxiety, depression, post-traumatic stress disorder (PTSD), and long-term trauma, while also undermining their ability to cope with uncertainty [3].

Eventually, Yasir secured admission to McMaster University’s Master’s in Global Health program through the Scholars at Risk Bursary (SARB) [4]. However, administrative delays to obtain a Canadian visa further delayed his education, prolonged feelings of instability and uncertainty, and exacerbated emotional strain. While grateful for the SARB program, Yasir’s experience highlights the systemic barriers refugee youth face in accessing higher education and employment. These often lengthy bureaucratic processes take a mental toll, leaving refugee youth feeling adrift and uncertain about their futures.

Globally, only 7% of refugee youth access higher education, compared with 40% of non-refugee youth [5]. Some initiatives, such as the Albert Einstein German Academic Refugee Initiative (DAFI), McMaster University’s SARB program, and Johns Hopkins University’s Peter Salama Refugee Scholarship, provide critical support but remain limited [6,7,8]. Expanding access to higher education for refugees is essential, not only for individual development, but also for protecting mental health by fostering hope, stability and long-term aspirations. Furthermore, improved educational access also contributes to the economic development and prosperity of host communities, representing multi-level benefits.

Yasir graduated from McMaster University in November 2023. Like many resettled refugees in high-income countries, he struggled to secure meaningful employment in his field of study—a challenge associated with feelings of isolation, low self-esteem, and depression among displaced youth [3]. Fortunately, his Mentors connected him to a research group focused on migrant and refugee health, leading to his current role as a research associate at a university program affiliated with UNHCR’s Group of Friends of Health [9]. A sub-group within this team, comprising eleven academic institutions, specifically aims to increase pledges from academic institutions for refugee youth scholarships [9].

While expanding scholarships for refugee youth is commendable, it does not guarantee successful integration in host countries. Scholarships alone offer limited support for refugee youth navigating unstable job markets and facing financial constraints in areas such as housing. Tailored mentorship programs after graduation are crucial to reinforce self-efficacy, build social support networks, and strengthen refugees’ sense of belonging. Existing evidence affirms that mentorship helps mitigate mental health risks for refugees, buffers stress and promotes psychological resilience [3].

Yasir’s journey from Kabul to Canada illustrates the transformative impact of combining scholarships, education, and mentorship for refugee youth’s success and well-being. Each component is necessary but insufficient alone; together, they enable healthy integration, creating a virtuous cycle where established refugees support host societies, including new arrivals. Further, once able, many refugees contribute to their countries of origin, strengthening transnational ties and fostering broader global development. Yasir’s story underscores the benefits of collective, cross-sectoral support across educational institutions, mentorship networks, and inclusive policies.

We propose four key recommendations to strengthen refugee youth success and well-being in host countries:

First, universities must design programs that accommodate refugees, including those without official documentation. The University of Ottawa’s Refugee Hub and Bard College’s global initiatives show how flexible admission policies can dismantle access barriers, restoring hope and promoting well-being [10,11].

Second, support must extend beyond scholarships to include post-graduation mentorship and career coaching. Refugees face cultural, linguistic, and systemic barriers that can lead to isolation, low self-esteem, loss of confidence and increased risk of depression and anxiety. Without structured mentorship, they risk incomplete integration after significant time and financial investment into their education or training. This unnecessarily limits refugee youth’s contributions and wastes preceding investments.

Third, credential recognition must be strengthened. Refugees possess valuable qualifications, yet many struggle with degree recognition, blocking professional advancement and leading to frustration, identity loss, and deteriorating mental health. Organizations such as the Canadian Information Centre for International Credentials and World Education Services have improved credential assessment, but more flexible systems are needed. Strengthening credential recognition frameworks would allow refugees to resume careers or transition into alternative pathways [12,13].

Finally, addressing these issues requires coordinated, multi-sectoral efforts. The Global Compact for Migration, adopted by 164 nations in 2018, emphasizes education and labor market integration for migrants and refugees. Civil society, academic institutions, and governments must collaborate in these efforts to ensure holistic support for displaced youth. The Global Refugee Sponsorship Initiative (GRSI), a Canadian-led model, demonstrates how community-driven programs can successfully enhance refugee integration and social cohesion [14,15].

Investing in refugees is not charity but a strategic investment that yields substantial economic and social benefits for host communities. Countries that support refugee youth succeed secure long-term gains. Modest reforms such as streamlining visa processes, reducing bureaucratic barriers, and expanding mentorship programs would not only enhance integration and uphold human rights, but also reduce psychological burden and promote well-being.

For example, 23% of refugees resettled in Canada earn a middle-class income, comparable to the national average [16]. Over 50% work in high-skilled professions, and approximately 20% hold positions requiring university degrees, including physicians, engineers, and architects [16]. Their integration not only enriches the cultural fabric of host societies, but also reinforces public services through lifetime tax contributions—further illustrating that inclusive approaches benefit all.

For 12 years, forced displacement has continued to rise unabated, with climate change expected to drive further displacements. As Khaled Hosseini reminds us, refugees share the same aspirations for safety, belonging and opportunity as others. Yet structural barriers persistently prevent them from rebuilding their lives and contributing fully to society. A comprehensive framework integrating scholarships, educational access, mentorship, psychological support, and employment pathways is critical to improving the health and well-being of refugee youth. Implementing these measures will not only empower displaced youth but also foster global resilience, prosperity, and mental health in the face of future displacement crises.
